# Survival of Proper Hepatic Artery Lymph Node Metastasis in Patients with Gastric Cancer: Implications for D2 Lymphadenectomy

**DOI:** 10.1371/journal.pone.0118953

**Published:** 2015-03-13

**Authors:** Cai Shirong, Chen Jianhui, Chen Chuangqi, Wu Kaiming, Zhang Xinhua, Song Wu, He Yulong

**Affiliations:** 1 Division of Gastrointestinal Surgery, The First Affiliated Hospital of Sun Yat-sen University, Guangzhou, China; 2 Gastric Cancer Center, Sun Yat-sen University, Guangzhou, China; Shanghai Jiao Tong University School of Medicine, CHINA

## Abstract

**Background and Aims:**

There is a discrepancy between the American Joint Committee on Cancer (AJCC) guidelines (7^th^ edition) and the Japanese treatment guidelines (3^rd^ edition) with regard to the extent of D2 lymphadenectomy for gastric cancer. In the AJCC, hepatic artery station (No.12a) lymph node (LN) metastasis is classified as distant metastasis, whereas in the Japanese guidelines, this classified is regional metastasis. This study aimed to evaluate whether it is appropriate to reclassify No.12a LN metastasis as distant metastasis in consideration of survival outcome.

**Methods:**

In this retrospective analysis, data from patients with gastric cancer who underwent regular D2 or greater lymphadenectomy between 1996 and 2006 were evaluated to determine any association between the clinicopathological features of hepatic artery LNs and survival prognosis.

**Results:**

Among the 247 patients with gastric cancer who underwent No.12 LN harvest, 45 (18.2%) were positive for No.12a LN metastasis. No.12a LN metastasis was significantly associated with poor clinicopathological features, advanced tumor stage, and poor overall survival. The 5-year survival rate of patients with No.12a LN metastasis was significantly better than that of patients with distant metastasis (*P* < 0.05), but was similar to that of patients with LN involvement in the D2 lymphadenectomy region (*P* > 0.05). No.12a LN metastasis was shown to significantly influence survival outcome in univariate analysis, but was not identified as a significant independent predictor in multivariate analysis. In logistic multivariate regression analysis, T stage, N stage, and station No.3, 5, and 6 LN metastasis were independent predictors of No.12a LN involvement.

**Conclusions:**

It is inappropriate to reclassify No.12a LN metastasis as distant metastasis. We propose that this be considered as regional metastasis and be included in the extent of D2 lymphadenectomy to improve survival outcomes in patients with gastric cancer.

## Introduction

The 7^th^ edition of the American Joint Committee on Cancer (AJCC) guidelines [1–2] for gastric cancer include many controversial changes that have been strongly debated, such as the restaging of gastric cancer and the reclassification of adenocarcinomas of the esophagogastric junction as esophageal carcinomas [3–6]. However, little attention has been given to the change that the station No.12a lymph nodes (lymph nodes along the proper hepatic artery) are no longer assigned to the D2 lymphadenectomy region and No.12a metastasis is reclassified accordingly as distant metastasis. Both the 6^th^ edition of AJCC guidelines [7] for gastric cancer and the 3^rd^ edition of Japanese treatment guidelines for gastric cancer [8] consider No.12a LN metastasis as regional metastasis from a primary gastric cancer that should be dissected during D2 lymphadenectomy to improve patient outcome. Moreover, most surgeons [9–10] agree that D2 lymphadenectomy is associated with an increased survival benefit in patients with gastric cancer. The differences between the guidelines may cause confusion among surgeons. The aim of this study was to evaluate the clinical importance and survival outcomes of patients with gastric cancer with No.12a LN metastasis and the difference in the efficacy of D2 lymphadenectomy according to the inclusion of metastatic No.12a LNs.

## Methods

The study was approved by the Institutional Review Board of the 1st Affiliated Hospital of Sun Yat-Sen University and informed consent was obtained according to institutional regulations. Written informed consent for further clinical research was given by participants for their clinical records to be used when patients were admitted to hospital.

Data obtained from patients with gastric cancer who underwent gastrectomy plus D2 or greater lymphadenectomy between January 1996 and December 2006 at the 1st affiliated hospital of Sun Yat-sen University were retrospectively analyzed. Patients who received neoadjuvant chemotherapy or chemoradiotherapy were excluded. The D2 lymphadenectomy region was determined according to the 6th edition AJCC gastric cancer guidelines and included the LNs along the left gastric artery (station No.7), the front of the common hepatic artery (station No.8a), the celiac axis (station No.9), the splenic hilum (station No.10), the splenic artery (station No.11), and the proper hepatic artery (station No.12a).

The clinicopathological features analyzed included age, sex, tumor size, histological tumor type, gross tumor type, tumor location, and cancer embryonic antigen (CEA) level. The histological type was defined as well or poorly differentiated. Poorly differentiated types included poorly differentiated adenoma, mucinous gastric cancer, signet ring cell cancer, and undifferentiated adenoma. The postoperative pathological stage was reclassified according to the 7^th^ edition of the AJCC gastric cancer guidelines.

　 Follow-up was conducted every 3–6 months for the first 3 years and once a year thereafter. Patients underwent regular blood tests including a tumor biomarker assessment, endoscopic examination, abdominal computed tomography or ultrasonography, and chest radiography or thoracic computed tomography. The last follow-up date was in December 2013. The mean follow-up time was 41.28 ± 34.84 months.

The chi-square test was used to compare differences in the categorical data. Survival was compared using the log-rank test, and survival curves were generated using the Kaplan-Meier method. The Life-table was used to calculate survival time. Univariate and multivariate analyses were conducted using the Cox proportional hazards regression model and forward logistic regression. Logistic regression analysis was used to determine factors associated with No.12a LN metastasis. A P-value <0.05 was considered statistically significant. All data were analyzed using the Statistical Package for Social Sciences (SPSS 16.0, Chicago, IL, USA).

## Results

### 1. Basic information

A total of 811 cases were eligible for study inclusion. Among the 247 patients with gastric cancer who underwent No.12 LN harvest, 45 (18.2%) were positive for No.12a LN metastasis. The mean number of No.12a LN metastases was 1.71 ± 1.84 (range: 1–11). To investigate the clinical significance of No.12a LN metastasis, clinicopathological characteristics were compared between cases with or without No.12a LN metastasis. Patients with No.12a LN metastasis had a large tumor size, a poor tumor histological and gross type, an abnormally high CEA level, deep tumor invasion, and extensive LN involvement (Table 1).

**Table 1:** Comparison of clinicopathological parameters in patients with or without No.12a station lymph nodes metastasis.

	No.12a(-)	No.12a(+)	*P* value
Age			0.857
≤60 year	415(54.2%)	25(55.6%)	
>60 year	351(45.8%)	20(44.4%)	
Gender			0.264
male	521(68.0%)	27(60.0%)	
female	245(32.0%)	18(40.0%)	
Tumor location			0.365
Upper 1/3	230(30.0%)	12(26.7%)	
Middle 1/3	154(20.1%)	6(13.3%)	
lower1/3	382(49.9%)	27(60.0%)	
Tumor diameter			0.003
≤5cm	431(56.3%)	15(33.3%)	
>5cm	335(43.7%)	30(66.7%)	
Gross type			0.003
Borrmann I+II	247(32.2%)	5(11.1%)	
Borrmann III+IV	519(67.8%)	40(88.9%)	
Histologic grade			0.025
G1+G2	240(31.3%)	7(15.6%)	
G3+G4	526(68.7%)	38(84.4%)	
T stage(7^th^)			<0.001
T1	110(14.4%)	0(0.0%)	
T2	80(10.4%)	0(0.0%)	
T3	131(17.1%)	1(2.2%)	
T4	445(58.1%)	44(97.8%)	
N stage (7^th^)			<0.001
N0	443(57.8%)	0(0.0%)	
N1	92(12.0%)	3(6.7%)	
N2	103(13.4%)	6(13.3%)	
N3	128(16.7%)	36(80.0%)	
Surgical prodecure			0.953
Distal gastrectomy	421(55.1%)	25(55.6%)	
Total gastrectomy	343(44.9%)	20(44.4%)	
CEA level			0.024
≤5mg/ml	693(90.5%)	36(80.0%)	
>5mg/ml	73(9.5%)	9(20.0%)	

### 2. Influence factors of No.12a LN metastasis

Univariate logistic regression analysis revealed that tumor diameter, histological type, gross classification, T stage, N stage, and CEA level were associated with No.12a LN metastasis (Table 2). In addition, the status of all LN stations included in the D2 dissection area, except for station No.2 LNs, influenced No.12a LN metastasis. Multivariate logistic regression analysis indicated that T stage, N stage, and No.3, 5, and 6 LN metastasis were independent predictors of No.12a LN involvement (Table 3).

**Table 2:** Univariate logistic regression analysis of the No.12a station lymph nodes metastasis.

	χ^2^ value	OR value	95% CI	*P* value
Age	0.033	-	-	0.857
Gender	1.246	-	-	0.264
Tumor location	1.017	-	-	0.313
Tumor diameter	8.483	2.573	1.362–4.861	0.004
Gross classification	7.738	3.807	1.484–9.766	0.005
Histological classification	4.695	2.477	1.090–5.627	0.030
UICC T stage	7.553	14.249	2.143–94.757	0.006
UICC pN stage	42.033	4.776	2.977–7.663	<0.001
No.1 (+)	5.222	2.240	1.122–4.472	0.022
No.2 (+)	3.235	-	-	0.072
No.3 (+)	17.732	3.710	2.016–6.830	<0.001
No.4 (+)	16.974	3.897	2.040–7.443	<0.001
No.5 (+)	48.476	9.511	5.045–17.930	<0.001
No.6 (+)	15.726	3.510	1.887–6.528	<0.001
No.7 (+)	14.735	3.618	1.876–6.977	<0.001
No.8a (+)	17.102	4.309	2.156–8.610	<0.001
No.9 (+)	18.325	7.655	3.015–19.438	<0.001
No.10 (+)	8.577	4.662	1.664–13.064	<0.001
No.11 (+)	27.081	9.829	4.156–23.244	<0.001
CEA level	4.850	2.373	1.100–5.122	<0.001

**Table 3:** Multivariate logistic regression analysis of the No.7 station lymph nodes metastasis.

	X^2^ value	OR value	95% CI	P value
Tumor invasion depth	4.699	8.060	1.221–53.182	0.030
Lymph node stage	31.495	4.210	2.549–6.956	<0.001
No.3 station lymph nodes metastasis	6.133	0.387	0.182–0.820	0.013
No.5 station lymph nodes metastasis	11.542	4.569	1.902–10.976	0.001
No.6 station lymph nodes metastasis	4.558	0.399	0.171–0.927	0.033

### 3. The survival significance of No.12a LN

**Fig 1:**
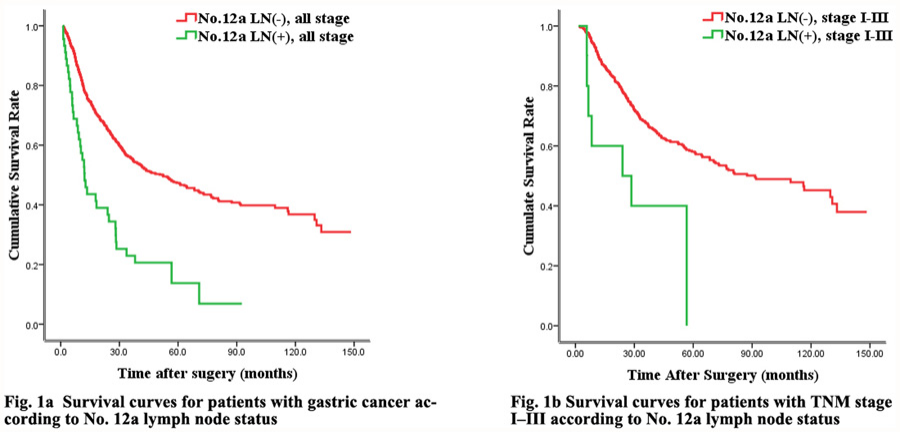
1a Survival curves for patients with gastric cancer according to No. 12a lymph node status. Fig. 1b Survival curves for patients with TNM stage I—III according to No. 12a lymph node status. The differences in the survival curves among the subgroups were statistically significant (*p* < 0.05).

A comparison of survival between patients with or without No.12a LN metastasis revealed that those with No.12a LN metastasis had a significantly poorer survival outcome (Table 4, Fig. 1a). A similar result was found between cases with or without No.12a LN metastasis in TNM I-III stages (Table 4, Fig. 1b). Moreover, although No.12a LN metastasis influenced the overall survival outcome, it was not an independent predictor (Table 5).

**Table 4:** The survival comparison of gastric cancer patients between No12a LN(+) and other stage.

	1ysr	3 ysr	5 ysr	*P* value	*Mst(m)*
Fig. 1a				<0.001	
No.12a LN(-), all stage	78.7%	55.7%	47.4%		50.3
No.12a LN(+), all stage	52.8%	23.0%	15.8%		14.4
Fig. 1b				0.021	
No.12a LN(-), TNM I-III stage	89.6%	67.7%	58.0%		88.5
No.12a LN(+), TNM I-III stage	70.0%	40.0%	20.0%		32.0
Fig. 2a				0.016	
No.12a LN(+), stage I-III	58.9%	28.0%	19.2%		21.5
All stage IV [including No.12a LN(+)]	39.9%	12.1%	7.9%		10.0
Fig. 2b				0.023	
No.12a LN(+), stage I-III	58.9%	28.0%	19.2%		21.5
No.12a LN(-), stage IV	40.7%	12.7%	8.3%		10.1
Fig. 2c				0.004	
No.12a LN(+), stage I-III	58.9%	28.0%	19.2%		21.5
No.12a LN(+), stage IV	25.0%	0.0%	0.0%		8.0
Fig. 2d				0.074	
No.12a LN(-), stage IV	40.7%	12.7%	8.3%		10.1
No.12a LN(+), stage IV	25.0%	0.0%	0.0%		8.0
Fig. 3a				<0.001	
N0	95.4%	77.6%	70.0%		136.9
No.12a LN(-), D1 LN(+)	91.0%	70.6%	51.9%		64.0
No.12a LN(-), D2 LN(+)	67.3%	28.6%	19.6%		21.5
No.12a LN(+), stage I-III	58.9%	28.0%	19.2%		21.5
No.12a LN(-), stage IV	40.7%	12.7%	8.3%		10.1
Fig. 3b				<0.001	
N0	95.4%	77.6%	70.0%		136.9
No.12a LN(-), D1 LN(+)	91.0%	70.6%	51.9%		64.0
No.12a LN(-), D2 LN(+)	67.3%	28.6%	19.6%		21.5
No.12a LN(+), all stage	52.8%	23.0%	15.8%		14.4
No.12a LN(-), stage IV	40.7%	12.7%	8.3%		10.1
Fig. 3c				0.518	
No.12a LN(+), stage I-III	58.9%	28.0%	19.2%		21.5
No.12a LN(-), D2 LN(+)	67.3%	28.6%	19.6%		21.5
Fig. 3d				0.119	
No.12a LN(-), D2 LN(+)	67.3%	28.6%	19.6%		21.5
No.12a LN(+), all stage	52.8%	23.0%	15.8%		14.4

**Table 5:** Univariate and multivariate Cox regression survival analysis of the gastric cancer.

	Univariate Cox regression analysis				Multivariate Cox regression analysis			
	χ^2^ value	OR	95%CI	*P* value	χ^2^ value	OR	95%CI	*P* value
Age	0.364	-	-	0.547				
Gender	0.201	-	-	0.654				
Tumor location	1.694	-	-	0.193				
Tumor diameter	113.577	2.871	2.365–3.486	<0.001	13.221	1.465	1.192–1.799	<0.001
Gross type	74.128	2.922	2.289–3.730	<0.001				
Histological type	16.356	1.559	1.257–1.934	<0.001				
T stage	143.363	2.370	2.058–2.730	<0.001	62.535	1.803	1.558–2.086	<0.001
N stage	84.221	1.428	1.323–1.541	<0.001				
M stage	276.781	5.570	4.550–6.820	<0.001	18.579	1.804	1.380–2.359	<0.001
No.12a	30.539	2.597	1.851–3.644	<0.001				
Radical surgery	318.418	6.723	5.453–8.288	<0.001	59.715	2.954	2.245–3.889	<0.001
CEA level	24.993	2.007	1.527–2.637	<0.001	9.828	1.556	1.180–2.051	0.002
Chemotherapy	2.054	-	-	0.152				

### 4. Survival comparison of No.12a LN metastasis and distant metastasis

**Fig 2:**
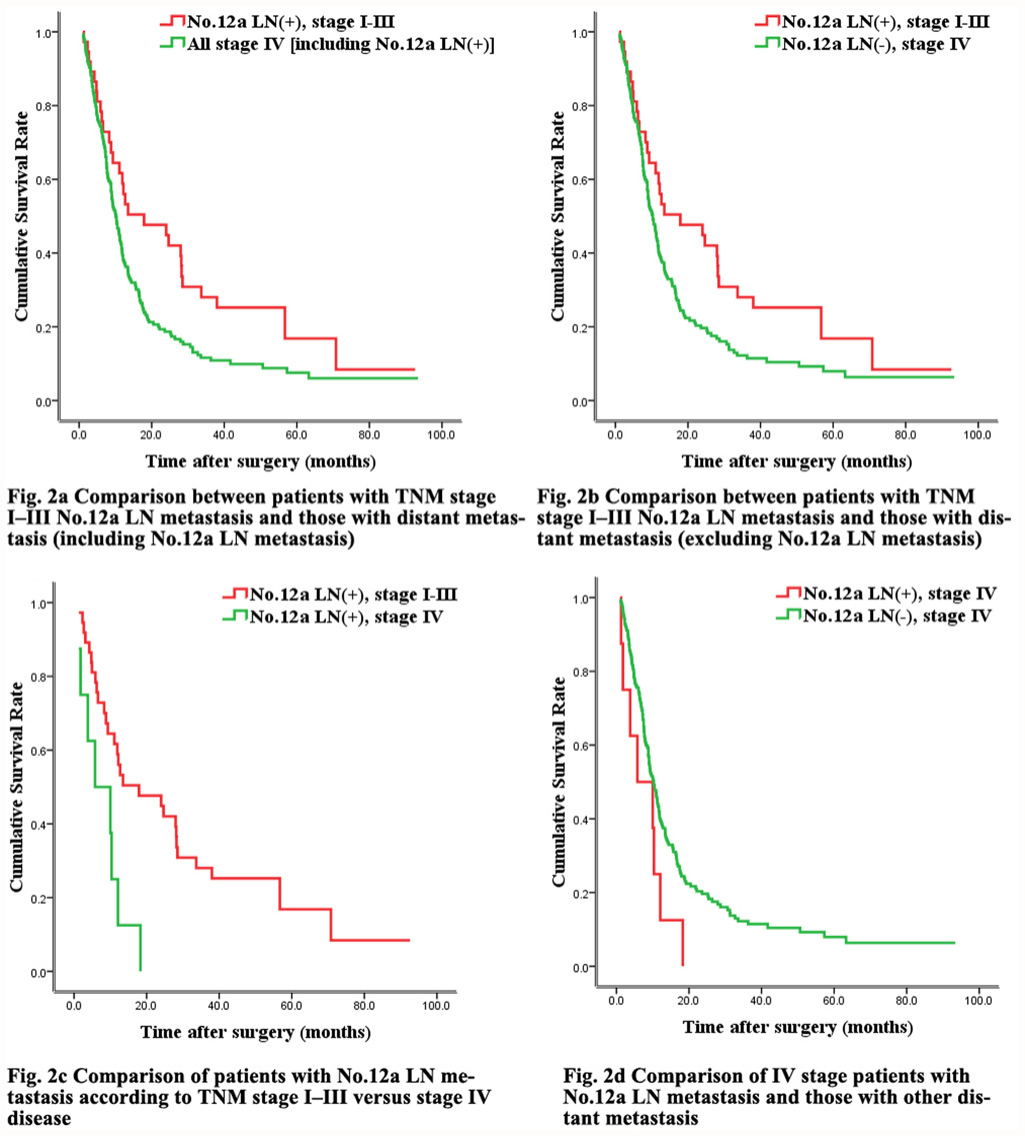
Overall survival rates in gastric cancer patients with No.12a lymph node metastasis versus distant metastasis. Fig. 2a Comparison between patients with TNM stage I–III No.12a LN metastasis and those with distant metastasis (including No.12a LN metastasis). The difference in survival curves was statistically significant (*p* < 0.05). Fig. 2b Comparison between patients with TNM stage I–III No.12a LN metastasis and those with distant metastasis (excluding No.12a LN metastasis). The difference in survival curves was statistically significant (*p* < 0.05). Fig. 2c Comparison of patients with No.12a LN metastasis according to TNM stage I–III versus stage IV disease. The difference in survival curves was statistically significant (*p* < 0.05). Fig. 2d Comparison of IV stage patients with No.12a LN metastasis and those with other distant metastasis. The difference in survival curves was not statistically significant (*p* > 0.05).

Since the 7^th^ edition of the AJCC guidelines for gastric cancer describes No.12a LN metastasis as distant metastasis, we compared the survival outcome between patients with No.12a LN metastasis and those with distant metastasis. Among the 45 patients with No.12a LN metastasis, 8 had distant metastasis. Interestingly, survival was significantly different between patients with TNM stage I–III No.12a LN metastasis and those with distant metastasis (including the 8 patients with No.12a LN metastasis). Distant metastasis was associated with a significantly worse overall survival than was No.12a LN metastasis among patients with stage I–III disease (Fig. 2a, Table 4). Similar results were obtained when survival was compared between patients with No.12a LN metastasis and those with distant metastasis excluding patients with No.12a LN metastasis, among patients with stage I–III disease (Fig. 2b, Table 4). In addition, the overall survival rate was significantly different between patients with TNM stage I–III No.12a LN metastasis and those with stage IV No.12a LN metastasis (Fig. 2c, Table 4). Finally, the overall survival rate of patients with TNM stage IV No.12a LN metastasis was lower than that of patients with distant metastasis, although the difference was not statistically significant (Fig. 2d, Table 4).

### 5. Survival comparison of No.12a LN metastasis and LN involvement in other lymphadenectomy regions

**Fig 3:**
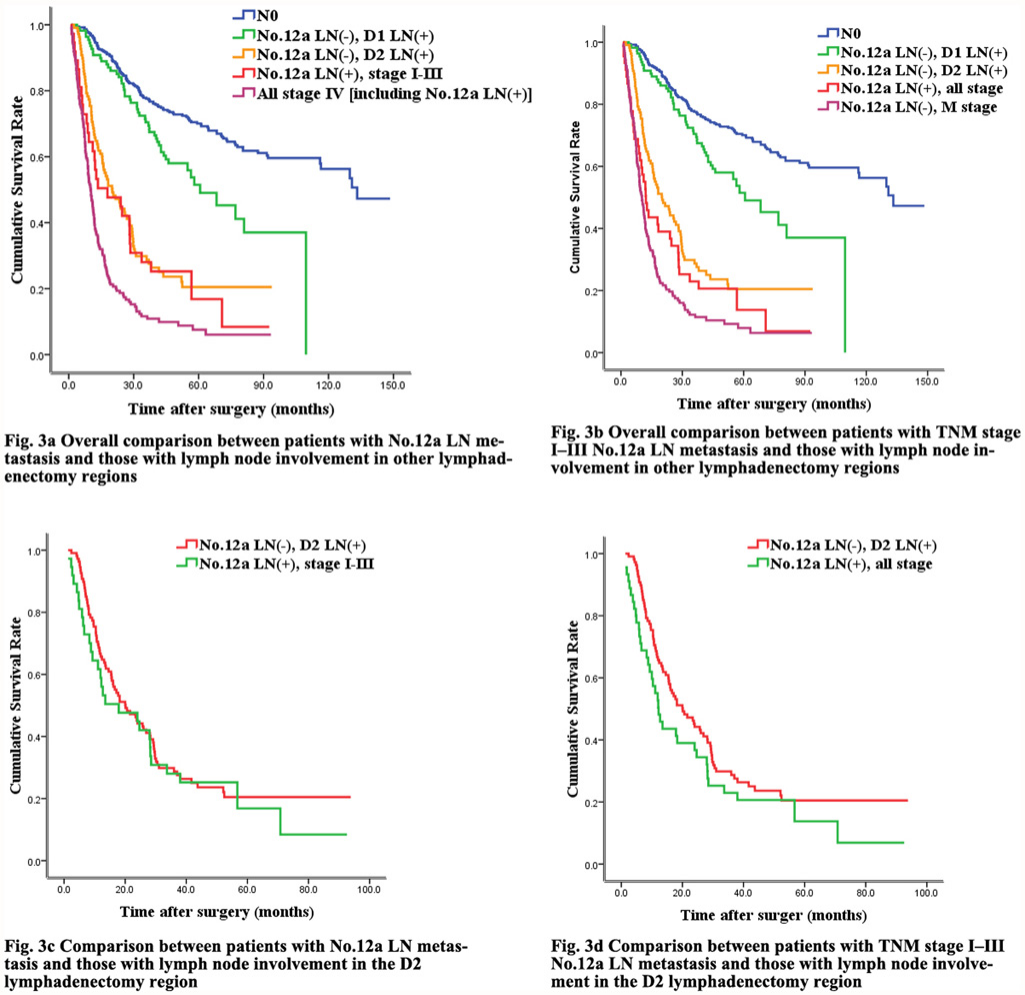
Overall survival rates for No.12a lymph node(LN) metastasis versus LN involvement in other lymphadenectomy regions. Fig. 3a Overall comparison between patients with No.12a LN metastasis and those with lymph node involvement in other lymphadenectomy regions. The difference in survival curves was statistically significant (*p* < 0.05). Fig. 3b Overall comparison between patients with TNM stage I–III No.12a LN metastasis and those with lymph node involvement in other lymphadenectomy regions. The difference in survival curves was statistically significant (*p* < 0.05). Fig. 3c Comparison between patients with No.12a LN metastasis and those with lymph node involvement in the D2 lymphadenectomy region. The difference in survival curves was not statistically significant (*p* > 0.05). Fig. 3d Comparison between patients with TNM stage I–III No.12a LN metastasis and those with lymph node involvement in the D2 lymphadenectomy region. The difference in survival curves was not statistically significant (*p* > 0.05).

Considering the significant difference in survival between patients with No.12a LN metastasis and distant metastasis, we compared survival data between patients with No.12a LN metastasis and those with metastasis in different lymphadenectomy regions to determine whether the No.12a LNs should be included in D2 lymphadenectomy. Irrespective of distant metastasis, there was a clear significant difference in overall survival among in patients with No.12a LN metastasis according to the status of different lymphadenectomy regions (Fig. 3a–b, Table 4). Moreover, we found that the differences between patients with No.12a LN metastasis and LN metastasis in the D2 dissection region were similar (Fig. 3c, Table 4). Similar results were obtained when comparing cases of No.12a LN metastasis excluding those of distant metastasis and cases of LN metastasis in the D2 dissection region (Fig. 3d, Table 4).

## Discussion

Surgery remains the only radical treatment option for patients with gastric cancer [11]. After the optimistic results of a Dutch clinical trial with a 15-year follow-up [12], D2 lymphadenectomy was widely accepted as the standard surgical procedure for advanced gastric cancer. Currently, there are 2 main staging systems for gastric cancer: the AJCC guidelines and the Japanese treatment guidelines. The AJCC guidelines are used worldwide, but most East Asian countries use both guidelines. Regional differences in gastric cancer are existed between Asian and Western countries with respect to etiology, prevalence, clinicopathological features and treatment strategy of the disease [13]. Hence some guidelines of the 7th edition UICC/AJCC maybe not suitable for the Asian countries. Both guidelines extol the benefits of D2 lymphadenectomy for the treatment of gastric cancer, but recent editions propound differing opinions in this regard. For example, in the latest edition of the Japanese treatment guidelines, the range of LN dissection is no longer based on the location of gastric cancer, but is decided according to the type of gastrectomy, where both the 6^th^ and 7^th^ editions of the AJCC guidelines for gastric cancer base the extent of D2 lymphadenectomy on tumor position. Moreover, in the 7^th^ edition of the AJCC guidelines for gastric cancer, the exclusion of No.12a LN dissection from D2 lymphadenectomy, was not explained, and may result present challenges when comparing past and present data as well as data between Eastern and Western countries.

Lee et al [14] reported that prognosis differed between patients with hepatoduodenal (No.12) LN metastasis and those with distant metastases. Similarly, in this study, survival outcomes were similar between cases of No.12a LN metastasis and those of LN involvement in the AJCC (7^th^ edition)-defined D2 lymphadenectomy region, although survival was poorer in cases of No.12a LN metastasis than in those of distant metastasis. No.12a LN metastasis in the current study was associated with poor malignant tumor behavior and advanced tumor stage. Moreover, No.12a LN metastasis was associated with poor overall survival in patients with gastric cancer. Although the LNs along the bile duct (12b) and portal vein (12p) are excluded from the extent of regular D2 lymphadenectomy, these results support the notion that the No.12a LN metastasis should be considered as regional LN metastasis.

Limitations of the current study include its retrospective design. Therefore, this study could not address the likelihood of station No. 12a nodal metastasis in patients deemed candidates for D2 dissection. A prospective randomized controlled study is needed in the future.

In conclusion, the survival outcome of patients with No.12a LN metastasis is similar to that of patients with LN involvement in the D2 lymphadenectomy region and worse than those of patients with distant metastasis, suggesting that the No.12a LN should be included in the definition of the D2 lymphadenectomy region in the gastric cancer guidelines.
